# Editorial: Knocking on neuroimmunology’s doors: an entrechat concerning the immune system balance and its cell metabolism orchestration

**DOI:** 10.3389/fimmu.2023.1236217

**Published:** 2023-06-26

**Authors:** Manolo Sambucci, Valérie Dardalhon, Daniela Latorre

**Affiliations:** ^1^ Neuroimmunology Unit, Santa Lucia Foundation IRCCS, Rome, Italy; ^2^ Institut de Génétique Moléculaire de Montpellier, Université de Montpellier, Centre National de la Recherche Scientifique (CNRS), Montpellier, France; ^3^ Institute of Microbiology, ETH Zurich, Zurich, Switzerland

**Keywords:** neuroimmunology, multiple sclerosis, immune system balance, myasthenia (myasthenia gravis - MG), narcolepsy type I

The physiological state of health of organisms depends on a fine tuning of immune activation and immune regulation pathways. Immune cells interact millions time a day with external environmental factors but also with symbiotic microorganism. It is a highly complex task for the immune system to address specific responses limiting host damage. The balance is dynamic but the mechanisms relevant for the immune-based disorders are not well understood. An example of such dynamic, combination of factors and events involved in the development of immune-based disease is provided by central nervous system (CNS) autoimmune disorders, arising neuroimmunology as field of study.

This Research Topic collects contributions addressing new evidence on the fundamental role of the immune system’s balance as key element in the crosstalk with the nervous system, with a particular focus on demyelinating disorder such as multiple sclerosis (MS) as well as on other brain-related autoimmune disorders such as narcolepsy type I (NT1) or disorders of the neuromuscular junction such as myasthenia gravis (MG).

MS is a chronic, inflammatory, demyelinating and neurodegenerative disease of the CNS. Although the causes of MS are still unknow, the involvement of an abnormal immune response, including the existence of autoreactive T cells and defects in T regulatory functions, is key in the pathophysiological mechanism of disease (1, 2). Here, Kunkl et al. reviewed the topic from a different perspective, summarizing current knowledge of the contribution of pathogenic T helper cells (Th) subsets on the astrocytic changes and altered behaviour observed in MS.

T cell polarization to either proinflammatory or regulatory cells is also influenced by metabolism. Several bodies of evidences in fact are showing that changes in nutrient availability can alter T cells function, giving particular attention to cellular metabolic remodelling in neurological disease (3–5). Pompura et al. reviewed current evidence on metabolic requirements of CD4^+^ T cell subsets as well as the role of lipids in T cell function, focusing on how the changes in lipid profile observed in patients with MS may influence the inflammatory functional phenotypes of T cells in the disease. Regarding the CNS and immune regulation, accumulating evidence suggests that circular RNA (circRNAs) could have an active role (6). Mycko et al. provided new insights into the role of circRNAs in the pathomechanism of MS, identifying two circRNAs and a group of miRNAs that are differentially expressed in MS patients and correlated with the disease state and severity, thus opening new venues for novel potential biomarkers and treatments of the disease. Remaining in the context of intrinsic factors underlying MS pathophysiological mechanisms, Derakhshani et al. found an increase of Hemoglobin Subunit Delta (HBD) in MS patients, suggesting the involvement of oxidative stress in MS development. To conclude, several microbial infections have been implicated in the aetiology of MS (7, 8) and increasing evidence points to an association of COVID-19 with a broad range of neurological disorders (9). In this Research Topic, MacDougall et al. reviewed the state of art regarding SARS-CoV-2 infection and MS, focusing on disease exacerbation and proposing an interesting model for how SARS-CoV2 can potentially shape CNS autoimmunity.

In this Research Topic, Meng et al. provide new insights on the immunomodulatory role of type 1 regulatory T cells (Tr1) in generalized myasthenia gravis. Moreover, Moschetti et al. analyzed T cell infiltrates in intracranial aneurysm (IA), describing the existence of T cells endowed with pro-inflammatory features as well as of Eomes^+^ Tr-1-like T cells that failed to display an immunomodulatory phenotype.

Finally, in their original article, Ayoub et al. reveal an altered composition of the proteome in the cerebrospinal fluid of NT1 patients, showing a dysregulation in the complement system, thus further supporting converging evidence of an immune-mediated mechanism underlying the disease pathophysiology (10).

Altogether, these works provide an update on the state of art on the immune-mediated mechanisms in neurological diseases highlighting the interpretation of nervous system as “special immune-controlled site”. This novel information could help the development of more targeted therapies.

## Author contributions

MS conceptualized and write the original draft of editorial, review and editing. VD and DL review and editing. All authors contributed to the article and approved the submitted version.
